# Multiple stress fractures of the lower extremity in healthy young men

**DOI:** 10.1007/s10195-011-0156-9

**Published:** 2011-10-15

**Authors:** Hyun-Ju Choi, Hong-Man Cho

**Affiliations:** 1 Department of Internal Medicine, Daejeon Hankuk Hospital, Daejeon, Korea; 2 Department of Orthopedic Surgery, Daejeon Veterans Hospital, Daejeon, Korea

**Keywords:** Multiple, Healthy, Stress fractures

## Abstract

Stress fractures result from abnormal stresses imposed on normal bones by the continued and repeated actions of muscles or from normal stresses imposed on abnormal bones. The risk factors that can cause such stress fractures include excessive use, such as, in soldiers or athletes, nutritional deficiencies, and endocrine disorders. In addition, disease may arise from long-standing rheumatoid arthritis, osteoporosis, corticosteroid therapy, joint stiffness or contracture, or the correction of angular deformity. In these cases, stress fractures may occur in one area or multiple areas. However, no case of multiple stress fractures in a young man who was not a professional athlete and who had no stress fracture risk factor, such as, an endocrine disease, has been previously reported.

## Introduction

Stress fractures result from abnormal stresses imposed on normal bones by the continued and repeated actions of muscles or from normal stresses imposed on abnormal bones. After a case of stress fracture in metatarsal bones was first described by Breithaupt in 1955 [1], this condition has been reported in the bones of the lower limbs. The risk factors that can cause such stress fractures include excessive use, such as, in soldiers or athletes, nutritional deficiencies, and endocrine disorders. In addition, disease may arise from long-standing rheumatoid arthritis, osteoporosis, corticosteroid therapy, joint stiffness or contracture, or the correction of angular deformity. In these cases, stress fractures may occur in one area or multiple areas. Although cases of multiple fractures are not common, reported cases include a 13-year-old female patient with juvenile idiopathic arthritis [2], a young female runner with exercise-induced amenorrhea under conditions of food restriction [3], a long distance runner with stress fractures in both femurs [4], and a weightlifter with stress fractures in both ulnas [5]. However, no case of multiple stress fractures in a young man who was not a professional athlete and who had no stress fracture risk factor, such as, an endocrine disease, has been previously reported. Here two such cases are reported together with a literature review.

## Case report

### Case 1

The 27-year-old male patient had marched for about 3–4 h a day after entering a recruit training center. The degree of difficulty was no different for him than for his colleagues. Due to pain in the left knee joint at around 2 weeks after entering the training center, in the right knee joint after 3 weeks, and in the right hip joint after 4 weeks, his training was reduced to walking to and from the training site.

**Fig. 1:**
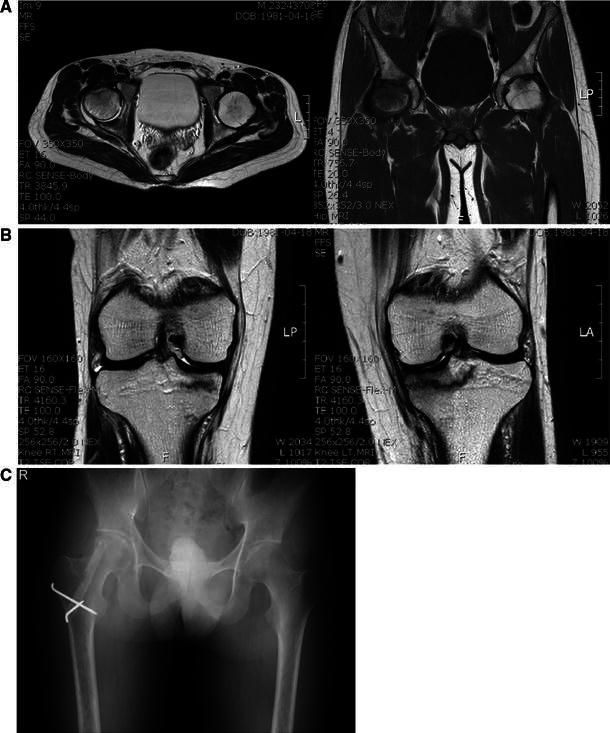
**a** The magnetic resonance imaging (MRI) obtained showed diffuse signal intensity (SI) decreases across the entire right head of the femur. **b** The magnetic resonance imaging (MRI) obtained showed that subchondral zones of low SI were observed in medial tibial plateaus on both sides. **c** Close reductions and internal fixations with fibular shaft graft and s-pins were performed on the subchondral stress fractures. At 2 years postoperatively, the patient still had considerable depressed deformation in the right femoral head

However, his symptoms did not improve and at 6 weeks after enrollment visited our hospital. A simple radiological examination failed to produce an abnormal finding, and thus, anti-inflammatory analgesics were prescribed and immobilization was recommended. When his symptoms were not improved 3 weeks after the first visit, magnetic resonance imaging was performed on the three areas where the patient reported pain (Fig. 1a). The magnetic resonance imaging (MRI) obtained showed diffuse signal intensity (SI) decreases across the entire right head of the femur and part of the right ischium, and in fat-saturated images, diffuse edematous images and subchondral zones of low SI were observed in medial tibial plateaus on both sides (Fig. 1b). Suspecting multiple stress fractures, we scrutinized patient history and performed a hematological examination.

The patient advised that he had grown in height until he was 22 years old. His body weight was 57 kg and height 162 cm, and there was no particular history of surgery or hospitalization. He had been eating small amounts of food since childhood, but was not a vegetarian and liked fish and meat. Seemingly, there was no finding to indicate a hormonal disorder, such as, abnormal findings with respect to early morning erection, sexual hair, etc., and a hematological examination showed a serum CTx (C-terminal telopeptide) 0.40 ng/ml, NTx (cross-linked N-telopeptides of type1 collagen in urine) 106 BCE/mM, 25(OH), Vitamin D3 20.9 ng/ml, PTH (intact) 23 pg/ml, LH 4.2 mIU/ml, FSH 3.7 mIU/ml, testosterone 2.63 ng/ml, osteocalcin 24.8 ng/ml, T3 (total) 119 ng/ml, free T4 1.34 ng/ml, TSH 1.40 uIU/ml, prolactin 3.4 ng/ml, alk phos 202 U/L, calcium 9.2 mg/dl, and phosphorus 4.2 mg/dl. Serum testosterone was low, but this did not match the results of his physical examination, and thus, was considered not of clinical significance. Dual energy X-ray absorptiometry (DEXA) showed a lumbar spine *T* score of 2.3. Based on the above mentioned MRI findings regarding medial tibial plateaus on both sides, conservative treatments, such as, long leg casting were performed.

A depression in the femoral head was found at simple radiographs 10 weeks after enrollment, and he was diagnosed with stress fractures of the right femur head. We decided to perform a vascularized fibular graft to stabilize the femoral head and prevent femoral head collapse.

Close reductions and internal fixations with fibular shaft graft and s-pins were performed on the subchondral stress fractures under spinal analgesia. The depression rate of the articular surface was low, and thus, to special treatment was given.

At 2 years postoperatively, the patient still had considerable depressed deformation in the right femoral head and the left medial tibial plateau. At this time the patient experienced pain and functional restriction, including restriction of dynamic ranges, and was walking with crutches (Fig. 1c).

### Case 2

The 28-year-old male patient had trained for about 2 weeks after entering a marine training center. It was said that 2-week training at the Marine training center consisted of 2–3 h of swimming training a day and 2 h of basic physical strength reinforcement training. The swimming training consisted of repetitions 50 min of swimming and 10 min of rest. The swimming portion consisted of swimming 25 m back and forth four times at full strength followed by swimming the same distance four times at low speeds. The trainees would then rest and begin again. The basic physical strength reinforcement training consisted of repeating walking and running for around 1 h followed by weight training to reinforce upper and lower extremity muscular strength. The degree of difficulty was no different for him than for his colleagues.

**Fig. 2:**
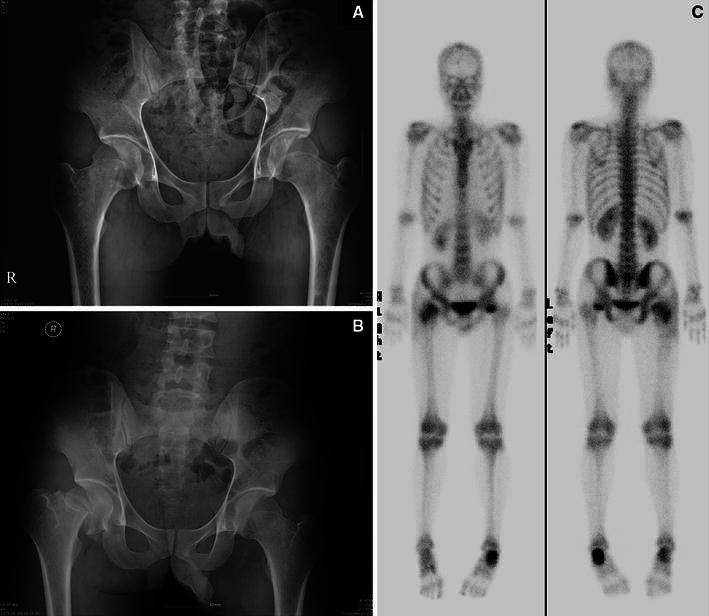
**a** A simple radiological examination was performed; no abnormal finding was found. **b** At 4 weeks after enrollment, a simple radiological examination was performed on the hip areas reported to be painful, and the radiographs obtained revealed a displaced femoral neck fracture. **c** A bone scan showed hot uptakes at both hips and at the left ankle joint

Due to pain in the both hip joints and the left ankle he visited a private clinic, but although a simple radiological examination was performed no abnormal finding was found (Fig. 2a); anti-inflammatory analgesics were prescribed and immobilization was recommended. At 4 weeks after enrollment, he presented at our hospital due to the aggravation of his right hip joint symptoms, a simple radiological examination was performed on the hip areas reported to be painful, and the radiographs obtained revealed a displaced femoral neck fracture (Fig. 2b). A bone scan showed hot uptakes at both hips and at the left ankle joint, MRI revealed fractures of both femoral necks (the right side was displaced and complete fracture) (Fig. 2c). He underwent open reduction and internal fixation at right hip using cannulated screws. The region of ankle joint hot uptake by bone scan was also investigated and found to be due to a slight fatigue fracture and articular depression of the subtalar joint and diffuse edematous change around the calcaneus on MRI. However, the patient has never made any specific complaint regarding the ankle joint. The left hip and ankle joint were treated conservatively by stabilization and long leg casting.

**Fig. 3:**
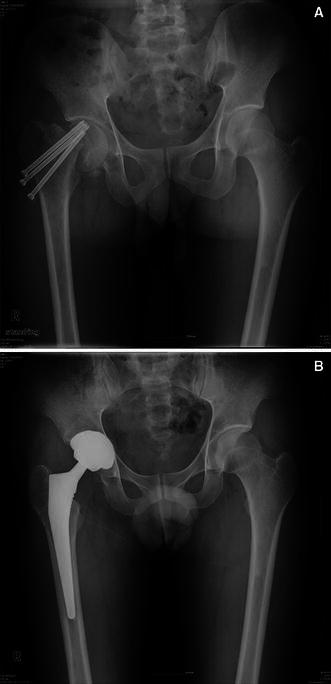
**a** Reduction loss was noted 2 weeks after surgery on the right hip. **b** The displaced femur neck fracture was treated by total hip arthroplasty

However, reduction loss was noted 2 weeks after surgery due to non-union (Fig. 3a) on right hip. The displaced femur neck fracture due to fracture non union was treated by total hip arthroplasty (Depuy pinnacle cup 54 mm, ceramic liner 54–36 mm, Corail stem 314, ceramic head 12/14 medium 36 mm) and operative findings were non-specific (Fig. 3b).

Suspecting multiple stress fractures, we scrutinized his history and performed a hematological examination.

The patient advised that he had grown in height until he was 21 years old. His body weight was 53 kg and height 170 cm, and there was no particular history of surgery or hospitalization. Seemingly, there was no finding to indicate a hormonal disorder, i.e., abnormal findings regarding early morning erection, sexual hair, etc., and dual energy X-ray absorptiometry (DEXA) returned a lumbar spine *T* score of 2.8. Findings from the hematological examination were a serum CTx (C-terminal telopeptide) 0.38 ng/ml, NTx (cross-linked N-telopeptides of type1 collagen in urine) 111 BCE/mM, 25(OH) Vitamin D3 22.3 ng/ml, PTH (intact) 27 pg/ml, LH 4.1 mIU/ml, FSH 3.7 mIU/ml, testosterone 2.79 ng/ml, osteocalcin 25.2 ng/ml, T3 (total) 121 ng/ml, free T4 1.35 ng/ml, TSH 1.42 uIU/ml, prolactin 3.5 ng/ml, alk phos 93 U/L, calcium 9.2 mg/dl, and phosphorus 4.4 mg/dl.

At 17 months postoperatively, the patient had a normal gait without any supportive equipment, and was pain free without functional restriction with stable fixated implants.

## Discussion

Stress fractures are caused by repetitive mechanical stresses and can be largely viewed as either fatigue or insufficiency fractures [6]. Fractures caused by repetitive mechanical stresses in healthy young or middle-aged patients are fatigue fractures, and stress fractures caused by low loads due to osteoporosis, osteomalacia, or other diseases in elderly patients are referred to as insufficiency fractures [7]. Overuse activities can increase the risk of multiple fatigue fractures in soldiers or untrained subjects. However, reports on multiple fatigue fractures thus far showed causes such as under-nutrition, dyscrinism, long-term rheumatoid arthritis, and osteoporosis. The two cases of multiple fatigue fractures that these authors investigated most likely resulted from overuse activities in soldiers and untrained subjects, as the authors observed that multiple fatigue fractures in three or more areas occurred in two healthy male patients without any under-nutrition, dyscrinism, long-term rheumatoid arthritis or osteoporosis.

Stress fractures occur in normal bones of healthy people without any particular trauma, and their types and locations depend on age and degree of activity. Regarding the mechanisms involved, Ingersoll [8] suggested that stress fractures occur in normal bones when fine fractures accumulate due to repetitive mechanical stimuli or external forces beyond the limits of skeletal maintenance. Belkin [9] reported bone resorption occurs and progresses as a reaction to stress and leads to fine fractures followed by complete fractures, and also reported that the frequency of stress fractures is increasing among athletes. However, Haider and Storey [10] advised that in patients with rheumatoid arthritis stress fractures were caused by systemic osteoporosis. Subsequently, stress fractures caused by long-standing rheumatoid arthritis have been well described. In addition, osteoporosis and corticosteroid therapy, which has the side effect of bone loss, are other known causes of stress fractures.

The areas affected by reported stress fractures show a predilection for metatarsal bones, but Belkin et al. [9] reported a high frequency in the tibia, and Green et al. [11] reported that the upper third of tibia was most affected. Risk factors for stress fractures revealed by epidemiological studies include sex (female), age, body composition, bone characteristics, low bone density and bone strength, low aerobic fitness, low past physical activity level, smoking, and excessive running [12]. If untoward cyclic loading is applied in the presence of these risk factors, stress fractures may occur even in young, healthy, and active individuals. However, the present cases did not have any predisposing factors.

Single stress fractures may occur in cases where there are no predisposing factors, and Bron et al. [13] reported single stress fractures in three healthy patients. However, cases of multiple stress fractures with no predisposing factors are rare, and are even uncommon in the presence of predisposing factors. Multiple stress fractures have been reported in patients with rheumatoid arthritis and in female patients with amenorrhea due to insufficient nutrition, excessive weight control, or excessive exercise. Cases of multiple stress fractures without such risk factors, include stress fractures in the cervical area of both femurs in a 19-year-old male soldier reported by Romero et al. [14], stress fractures in both femurs in a 19-year-old male soldier reported by Salminen et al. [12], and cases among professional athletes, such as, supracondylar stress fractures in both femurs in a 14-year-old cross country runner [15], and stress fractures in both ulnas in a weight lifter [5]. However, as yet no case of stress fractures in three or more areas has been described in a young, healthy male soldier.

In a case of stress fractures in the cervical areas of both femurs in a healthy 19-year-old male, Romero et al. [14] reported that the patient showed no large differences in degree of training or hematological or bone tissue examination findings as compared with his colleagues. Egol et al. [6] found that stress fractures in the cervical areas of the femurs occurred largely in two types of patient groups, that is, young, healthy, active individuals, such as, recreational runners, endurance athletes, and military recruits, and in elderly people with osteoporosis. They advised that the condition can largely be divided into two types: fatigue fractures caused by untoward cyclic loading or impaired bone quality, or insufficiency fractures. The present case could be said to have been caused by untoward cyclic loading in a young, healthy, active individual.

Insufficiency subchondral fractures in the femur, as occurred in our first case, are a rare cause of acute pain in the hip joint in healthy people, and most reported cases show little relation to other diseases, medication, or smoking [16]. These fractures frequently occur in patients of 60 years or older with a low Singh index and low bone density. In many cases, initial radiographic findings are normal, but flattening of the bone head progresses suddenly, which supports the hypothesis of Hagino and Okana et al. [17] that insufficiency subchondral fractures may induce rapid destructive arthropathy.

It is of considerable importance that insufficiency subchondral fractures, osteonecrosis, and transient osteoporosis be differentiated, because their treatments and prognoses are quite different. Of course, since the causes of individual cases differ, history taking of alcohol and steroid intake and others is important, but much scope remains for controversy regarding radiological diagnoses. Some have reported differential diagnoses being made based on MRI findings, and that insufficiency subchondral fractures are characterized by a low signal intensity serpiginous line paralleling the articular surface. Transient osteoporosis, which should be distinguished as such, occurs in young patients as a self-limiting syndrome, and focal bone losses or subchondral collapses are not apparent during radiological examinations [18], whereas MRI may show diffuse and homogeneously reduced signal intensities in T1-weighted images and increased signal intensities in T2-weighted images, but no localized lesions [19–22].

The most important factor regarding the treatment of stress fractures is early diagnosis. Therefore, it is considered patients that report severe pain in bones or joints after overuse should be actively examined, even in the absence of an abnormality in bone metabolism or of any risk factor thought to induce stress fractures, such as, endocrine disease.
